# The Multiplicity of Post-Translational Modifications in Pro-Opiomelanocortin-Derived Peptides

**DOI:** 10.3389/fendo.2013.00186

**Published:** 2013-12-02

**Authors:** Akikazu Yasuda, Leslie Sargent Jones, Yasushi Shigeri

**Affiliations:** ^1^Health Research Institute, National Institute of Advanced Industrial Science and Technology (AIST), Ikeda, Japan; ^2^Department of Biology, Appalachian State University, Boone, NC, USA

**Keywords:** CLIP, END, MALDI-MS, MSH, POMC, post-translational modification, topological mass spectrometry analysis

## Abstract

The precursor protein, pro-opiomelanocortin (POMC) undergoes extensive post-translational processing in a tissue-specific manner to yield various biologically active peptides involved in diverse cellular functions. The recently developed method of matrix-assisted laser desorption/ionization mass spectrometry (MALDI-MS) for direct tissue analysis has proved to be a powerful tool for investigating the distribution of peptides and proteins. In particular, topological mass spectrometry analysis using MALDI-MS can selectively provide a mass profile of the hormones included in cell secretory granules. An advantage of this technology is that it is possible to analyze a frozen thin slice section, avoiding an extraction procedure. Subsequently, tandem mass spectrometry (MS/MS) has a profound impact on addressing the modified residues in the hormone molecules. Based on these strategies with mass spectrometry, several interesting molecular forms of POMC-derived peptides have been found in the fish pituitary, such as novel sites of acetylation in α-melanocyte-stimulating hormone (MSH), hydroxylation of a proline residue in β-MSH, and the phosphorylated form of corticotropin-like intermediate lobe peptide.

## Introduction

It is now recognized that pituitary hormones such as melanocyte-stimulating hormone (MSH), corticotropin-like intermediate lobe peptide (CLIP), and endorphin (END), are processing products of a common precursor protein, pro-opiomelanocortin (POMC). This knowledge was discovered through traditional endocrine research and nucleotide sequencing of the cloned cDNA for the POMC precursor. In 1916, the first demonstrations that the pituitary is involved in controlling skin color were found when hypophysectomy of tadpoles caused a skin lightening, while injection of pituitary extracts produced skin darkening (1, 2). The development of assays was then devised to test the response to the melanocyte-stimulating factors (3), for control of the adrenal cortex (4), and for opiate-like actions (5). The strategy using the bioassay is the commonly accepted approach for the purification of pituitary hormones. Nakanishi et al. (6) first reported the nucleotide sequence of a cloned cDNA encoding bovine corticotropin and β-lipotropin precursor, which consists of repetitive units of melanotropin, CLIP, and END sequences. Electron microscopy was applied to pituitary studies in 1950s. These revealed that secretory proteins are temporarily stored within special storage granules and subsequently secreted into the blood stream. In the late 1960s, there were several attempts to isolate secretory granules in an application of ultracentrifugation, and then the bioassay and granule measurement supporting the ultrastructural identification of the pituitary cell types were reported by many investigators. After 30 years of these types of studies, matrix-assisted laser desorption/ionization with time-of-flight mass spectrometry (MALDI-TOF MS) was developed to analyze the secretory granules directly in frozen sections. In this review we shall focus on recent studies of the post-translational modifications that have been found in POMC-derived peptides in fish pituitary, starting with a background on the application of mass spectrometry that has been used for these studies.

## Direct Tissue Analysis Using MALDI-TOF MS

The origin of direct laser desorption/ionization for mass spectral analysis may be traced to the early 1960s (7). This technology evolved into the laser microprobe mass analyzer (LAMMA) as a tool for medical and biological samples that could be aimed at a region of interest in a histological microtome section (8). Interpretation of the LAMMA spectra subsequently led to the discovery of the MALDI principle in 1985 (9). Recently, MALDI imaging mass spectrometry (IMS) has emerged as a new method that allows mapping of a wide range of biomolecules within a thin tissue section [see, Ref. (10)]. Independently of, and parallel to IMS studies, we proposed “topological mass spectrometry analysis” (11) as a strategy based on an application of MALDI-TOF MS analysis that allows for the search for novel hormones and neuropeptides. A key benefit in our method of sample preparation is that, while the ion peaks generally could not be detected in the frozen slices of an endocrine gland or neuron prepared at a 30–40 μm thicknesses, significant signaling in the spectrum could be measured specifically from the secretory cells irradiated by the laser light. The resulting spectrum indicates that MALDI-TOF MS selectively provides a mass profile of the peptides and proteins derived from cell secretory granules. Our hypothesis is that selective detection of secretory granules seems to be caused by proton capture of granules during MALDI ion formation, and not by other cellular components. Another benefit of direct tissue MALDI-TOF MS is that any enzymatic event in the sample preparation is suppressed by the matrix solution, which possesses a strong inhibitory effect. Thus, instead of a bioassay, the resulting molecular weight becomes an index of hormone purification. In contrast, IMS peak intensity and the number of observed peaks are prominently increased by the thinness of a slice decreased to <10 μm, in which most of the ion peaks seem to be from substances of cytoplasmic origin. Topological mass spectrometry analysis comprises three steps. The first one is the site-specific molecular mass profiling of the peptides and proteins contained in the secretory granules by direct tissue analysis using MALDI-TOF MS. The identification of hormones is accomplished by matching the observed masses to the theoretical masses derived from a sequence database. Thus, the unidentified ion peaks in the spectrum may suggest the presence of a novel candidate hormone or a modified hormone in the pituitary. In the interpretation of the resulting spectrum, it is interesting that mass differences such as −1, 16, 42, and 80 Da correspond to carboxy-terminal amidation, oxidation, or hydroxylation, acetylation, and phosphorylation, respectively. This rule can be applied to predict the modified residue in a hormone. The second step is purification of a given peptide from the tissue extract by monitoring of the resulting mass number, and then sequencing the peptide using a protein sequencer or tandem mass spectrometry (MS/MS) analysis. The third step is molecular cloning to determine the precursor sequence corresponding to the isolated peptides. The liquid chromatography (LC) MS/MS analysis is also available to determine the mature molecules with post-translational modifications. Application of topological mass spectrometry analysis is widely used in the fields of endocrinology and neuroscience in fish, crustaceans, insects, and plants (12–20). Among them, the medaka pituitary provides a unique spectrum in direct MALDI-TOF MS that was the basis of the first report of the structural determination of tri-acetylated and hydroxylated POMC-related hormone in vertebrates (19).

## Acetylation

**Figure 1:**
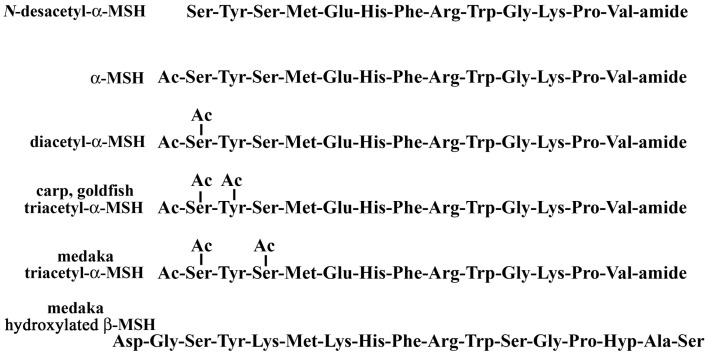
**Post-translational modifications of melanotropins in the pituitary**.

Acetylation is a major modification in POMC-derived peptides, such as α-MSH and END. α-MSH is mainly produced in the pituitary and controls melanosome dispersion in melanocytes. The structure of α-MSH is characterized as a tri-decapeptide including an amino-terminal acetylation and a carboxy-terminal amidation that is identical in most vertebrates. Structural variations of α-MSH were first found in bovine pituitary as the diacetyl form (21), and identified from salmon pituitary as the non-acetylated form (22). Recently, based on topological mass spectrometry analysis, we have reported that four molecular forms, *N*-desacetyl-, monoacetyl-, diacetyl-, and triacetyl-α-MSH, were present in medaka pituitary as shown in Figure 1 (19). Tandem mass spectrometry (MS/MS) analysis has shown that N,O-diacetylation of the Ser residue at position 1 was deduced from diacetyl-α-MSH, and N,O-diacetylation of that same Ser residue at position 1 and O-acetylation of the Ser residue at position 3 from triacetyl-α-MSH. In an earlier study on fish endocrinology, *N*-desacetyl-, monoacetyl-, and diacetyl-α-MSH were identified from two species of Cyprinidae: goldfish and carp (23). More recently, when we have checked pituitary further in carp and goldfish using direct MALDI-TOF MS, the existence of triacetyl-α-MSH was observed. However, in a comparison of triacetyl-α-MSH between medaka and carp/goldfish with MS/MS, the resulting spectra were different from each other. The N,O-diacetylation of the Ser residue at position 1 and O-acetylation of the Tyr residue at position 2 in the triacetyl-α-MSH molecule were deduced in carp and goldfish (20). The acetylation reaction of the α-MSH molecule starts with the amino group on the Ser residue at position 1, and then act with the hydroxyl group at the N-terminal Ser residue. A definitive O-acetylation for [*N,O*-diacetyl Ser^1^]-α-MSH may vary from species to species.

We have carried out a physiological study using a MALDI-TOF MS that directly analyzes and measures those hormones. The percentages as a total of α-MSH molecules were compared for medaka and goldfish reared in a white or black tank for 5 days. Among structural variants, diacetyl-α-MSH was the predominant form in goldfish and *N*-desacetyl-α-MSH in medaka, respectively. In both species, the relative level of the predominant form in the pituitary of white-adapted fish tended to be lower than that of black-adapted fish, but no significant difference was observed in the relative content of goldfish triacetyl-α-MSH in both backgrounds. Furthermore, the lowest content of triacetyl-α-MSH was found in black-adapted medaka. These preliminary data beg the question of how the physiological events regulate the variation in α-MSH levels or the different pattern of acetylation of α-MSH in the fish pituitary.

## Hydroxylation

Hydroxylation of the proline residue in the pituitary hormones has never been found previously. Recently, we reported the existence of a hydroxyproline (Hyp) residue in the β-MSH molecule in medaka pituitary (19). β-MSH is a slightly basic peptide and there are some variations between different species. A post-translational modification of the β-MSH molecule was not previously observed in any species. In fact, direct tissue MALDI-TOF MS analysis using tissue slices from carp, goldfish, rainbow trout, etc. indicated that β-MSH containing a Hyp residue was not present. Thus, β-MSH is an enigmatic hormone with hydroxylation of melanotropin, restricted to β-MSH in the medaka species, and a physiological role of β-MSH in teleost fish has not yet been elucidated. On the other hand, in mammalian and frog hypothalamus, an endogenous, hydroxylated post-translational product of the luteinizing hormone-releasing hormone sequence [Hyp^9^]-LHRH, has been isolated (24). In rodent, the [Hyp^9^]-LHRH may play a specific role in development, as [Hyp^9^]-LHRH is the major molecular form in the fetal rat hypothalamus, but is less potent than LHRH on gonadotropin secretion.

## Phosphorylation

**Figure 2:**
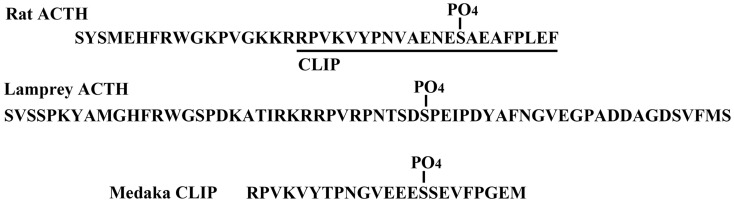
**Phosphorylation of ACTH and CLIP molecules in the pituitary**.

The structure of CLIP is known as a C-terminal fragment of the adrenocorticotropic hormone (ACTH) sequence. A phosphorylated form of CLIP has been identified in rat pituitary (25), in which a serine residue at position 14 is phosphorylated (*O*-pSer). The location of the *O*-pSer residue in medaka CLIP is in a similar position to that of the rat CLIP. In addition, similar to rat and human ACTH, the lamprey ACTH is partially phosphorylated at the Ser residue at position 35 as shown in Figure 2 (16). In comparing mammalian ACTH and CLIP, the sequence of -Ser-Ala-Glu- is part of the signal for possible phosphorylation of the Ser residue. In fish hormones, the sequence of -Ser-Ser-Glu- in medaka CLIP and sequence of -Ser-Pro-Glu- in lamprey ACTH are observed. In contrast, in porcine ACTH, a leucine residue is found at position 31 instead of a serine residue (26). So far, a phospho-ACTH or phospho-CLIP have not been found in porcine samples. Thus, a consensus sequence of Ser-X-Glu seems to be a signal of phosphorylation in the pituitary hormone. However, the physiological role of phosphorylation in ACTH and CLIP molecules as a primary messenger remains to be elucidated in any vertebrate species including the lamprey.

## Conclusion and Recommendations

Mass spectrometry has developed into the method for identifying proteins and elucidating their post-translational modifications, by using the amino acid sequence in a public database. Most POMC-derived peptides undergo some form of modification following translation. In recent studies, multiple forms of hormones with post-translational modifications, such as acetylation, hydroxylation, and phosphorylation, can be identified in fish pituitary. These modifications result in mass changes that are detected during analysis, such as topological mass spectrometry. As a result, the mass spectrometric analysis of these post-translational modifications is particularly important in endocrine research. Furthermore, direct tissue analysis using MALDI-TOF MS is an analyzable experimentation if there is one only pituitary and if there are just several minutes. Thus, this strategy is becoming an essential tool for endocrine researchers to elucidate molecular mechanisms within cellular systems. On the other hands, the post-translational modifications of hormone seem to be involved in diverse functions. However, the physiological significance of the modified hormones remains to be elucidated. It is, therefore, necessary to clear a physiologic role about the multiplicity of pituitary hormone in the future.

## Conflict of Interest Statement

The authors declare that the research was conducted in the absence of any commercial or financial relationships that could be construed as a potential conflict of interest.
